# Diamond Supercapacitors: Towards Durable, Safe, and Biocompatible Aqueous-Based Energy Storage

**DOI:** 10.3389/fchem.2022.924127

**Published:** 2022-05-20

**Authors:** Andre Chambers, Steven Prawer, Arman Ahnood, Hualin Zhan

**Affiliations:** ^1^ School of Physics, University of Melbourne, Parkville, VIC, Australia; ^2^ School of Engineering, RMIT University, Melbourne, VIC, Australia; ^3^ School of Engineering, Australian National University, Canberra, ACT, Australia

**Keywords:** diamond, supercapacitor, bioelectronic device, computational modelling, stability, large voltage window, biocompatible, aqueous electrochemical capacitor

## Abstract

Durable and safe energy storage is required for the next generation of miniature bioelectronic devices, in which aqueous electrolytes are preferred due to the advantages in safety, low cost, and high conductivity. While rechargeable aqueous batteries are among the primary choices with relatively low power requirements, their lifetime is generally limited to a few thousand charging/discharging cycles as the electrode material can degrade due to electrochemical reactions. Electrical double layer capacitors (EDLCs) possess increased cycling stability and power density, although with as-yet lower energy density, due to quick electrical adsorption and desorption of ions without involving chemical reactions. However, in aqueous solution, chemical reactions which cause electrode degradation and produce hazardous species can occur when the voltage is increased beyond its operation window to improve the energy density. Diamond is a durable and biocompatible electrode material for supercapacitors, while at the same time provides a larger voltage window in biological environments. For applications requiring higher energy density, diamond-based pseudocapacitors (PCs) have also been developed, which combine EDLCs with fast electrochemical reactions. Here we inspect the properties of diamond-related materials and discuss their advantages and disadvantages when used as EDLC and PC materials. We argue that further optimization of the diamond surface chemistry and morphology, guided by computational modelling of the interface, can lead to supercapacitors with enhanced performance. We envisage that such diamond-based supercapacitors could be used in a wide range of applications and in particular those requiring high performance in biomedical applications.

## Supercapacitors and the Allure of Diamond

Supercapacitors are a type of energy storage device which have been the focus of intense research over the past decades, having the potential to address the limitations in power density and lifetime of current rechargeable battery technology (Simon and Gogotsi, 2008). While high-energy-density rechargeable batteries operate via relatively slow reversible electrochemical reactions, supercapacitors store energy through electrostatic absorption/desorption and/or fast Faradaic mechanisms at the electrode-electrolyte interface. They thus offer higher power densities, i.e., delivering sufficient energy in a short time, shorter charging/discharging rates, and increased cycling stability. On the other hand, supercapacitors generally exhibit intermediate energy densities between rechargeable batteries and conventional double plate capacitors, and so are currently best suited for applications requiring short bursts of high power. These include hybrid transportation, regenerative breaking, portable and wearable electronics, and medical applications (Sinha and Kar, 2020).

Supercapacitors are generally classified into electrochemical double layer capacitors (EDLCs) and pseudocapacitors (PCs). While EDLCs store energy through electrostatic absorption of ions at the electrode-electrolyte interface, PCs utilize fast Faradaic mechanisms (redox reactions) to store energy. The absence of redox reactions in EDLCs leads to high cyclic stability but, due to the screening-affected electrostatic absorption, their energy density is limited. The two key performance metrics for supercapacitors are the energy (E) density and power (P) density. PCs based on materials such as conducting polymer and transition metal oxides typically possess a significantly greater E density (exceeding 100 Wh kg^−1^) compared to EDLCs such as activated carbon or graphene (on the order of 5–10 Wh kg^−1^), but often exhibit lower P density and poorer stability (Yu et al., 2018a). In the broader area of supercapacitor research, PCs are becoming favored over EDLCs because of this superior E density; however, EDLCs are still considered for small scale but high-power applications. The optimization of E and P densities relies on an electrode material with a large specific surface area (SSA), high porosity, high stability and conductivity. One must also consider the choice of electrolyte, with high conductivity, ion sizes that match the electrode pore sizes, and a wide electrochemical potential window (Sinha and Kar, 2020; Yu et al., 2021).

In particular, the advantage of a wide electrochemical potential window in aqueous electrolytes directly affects the supercapacitor performance, as both the E and P density are proportional to the square of the potential window (Gao and Nebel, 2016). This window can be widened by using ionic liquids, or organic and “water-in-salt” electrolytes (for example [EMIM][BF_4_], TEABF4/CAN, and LiTFSI, respectively); however, many of these chemicals are flammable or toxic (Martínez-Casillas et al., 2019). Highly toxic electrolytes present a significant health hazard for biomedical applications even if hermetically sealed, and so safer aqueous electrolytes are preferred (Raravikar et al., 2017). Aqueous electrolytes also have high conductivity at room temperature enabling higher P densities, while are generally less expensive than organic electrolytes or ionic liquids (Simon and Gogotsi, 2013; Gao and Nebel, 2016; da Silva et al., 2018). However, the potential window of conventional supercapacitor materials such as activated carbon in aqueous electrolytes is limited to approximately 1.2 V (−0.4 V to +0.8 V vs. SHE at pH = 7) (Kim et al., 2014). If this voltage is exceeded, oxygen and hydrogen evolution reactions take place, leading to the compositional change of the electrolyte and the corrosion of the electrode material (Keskinen et al., 2012; Wang et al., 2020a).

Diamond – particularly boron or nitrogen doped conductive diamond – has been viewed favorably as a supercapacitor electrode material primarily for its potential to address this issue, possessing a wide electrochemical potential window of up to 3.5 V in aqueous electrolyte to allow higher operation voltages (Gao and Nebel, 2016; Yu et al., 2017; Wang et al., 2020a). While diamond is not yet widely employed due to the relatively high cost and difficulty in processing (Siuzdak and Bogdanowicz, 2018; Yu et al., 2021), it nevertheless has attracted interest for its wide potential window and other excellent properties. For example, diamond is durable and biocompatible, making it suitable for long-term biomedical applications such as high capacitance neural interfaces (Hébert et al., 2014; Garrett et al., 2016; Tong et al., 2016; Siuzdak and Bogdanowicz, 2018; Wang et al., 2020a; Falahatdoost et al., 2021). Diamond also possesses high resistance to biofouling (Trouillon and O’Hare, 2010), and has demonstrated high capacitance in saline solution (Falahatdoost et al., 2021). Moreover, diamond is stable in corrosive chemicals such as nitric or sulfuric acid (Hoffmann et al., 2010), and is resistant to degradation through electrochemical oxidation in contrast to other sp^2^ carbon materials (Castanheira et al., 2014; Gao and Nebel, 2016). These properties suggest the use of diamond in surgically implantable supercapacitors, which are attractive power sources for biomedical devices due to high power density, long lifespan, and small dimensions (Chae et al., 2017; Sim et al., 2018; Liu et al., 2021). Diamond-based supercapacitors are able to use safer aqueous electrolytes while retaining an extended potential window, or could possibly even use the physiological electrolyte itself, as has been done previously with other materials [see (Chae et al., 2017; Raravikar et al., 2017; Sim et al., 2018)].

Here, we present our perspective on the current progress and challenges in the development of diamond-based supercapacitors. We argue that while existing diamond-based materials do not have the highest capacitance or energy storage compared with other materials, they nevertheless are well-suited to specialized applications, such as aqueous-based supercapacitors in biomedical electronics. In addition, we suggest diamond could be used in composite-material supercapacitor devices to enhance the electrochemical and environmental stability of existing PC materials. To further optimize diamond-based electrodes for these applications, we propose that special attention should be paid to both the surface chemistry and morphology of the diamond surface in relation to the chosen electrolyte. In particular, optimization of the nanoporous surface can be assisted by computer modelling of ionic processes within the pores of the electrode.

We have noted several past reviews on current research progress on diamond-based supercapacitors, which focus on summarizing the performance and fabrication methods of diamond-based supercapacitors (Gao and Nebel, 2016; Siuzdak and Bogdanowicz, 2018; Yu et al., 2021). In addition to these reviews, here we focus on the specific impacts of surface chemistry and morphology of the diamond surface, which would provide additional vital insights. Moreover, we discuss the biocompatibility aspect of diamond, which combined with its durability and wide potential window in aqueous electrolytes, makes it a promising candidate for long-term biomedical applications.

Below we briefly outline current experimental progress in the fabrication and application of diamond-based EDLCs and PCs, highlighting the importance of surface chemistry on device performance. We also discuss device level experimental questions which can be assisted by computational simulations, including the modelling of key parameters such as the capacitance in relation to the morphology of porous surfaces. We examine problems and progress with current modelling techniques for supercapacitors, and how this relates to diamond-based materials. We finally weigh up the advantages and disadvantages of diamond-based supercapacitors and offer our perspective on the future prospects and applications of these devices.

## Current Experimental Progress of Diamond Supercapacitors

Several different classes of diamond-based materials have been developed for supercapacitor applications. As mentioned above, supercapacitors can be divided into electrical double layer capacitors (EDLCs) and pseudocapacitors (PCs), each requiring different materials and properties. PCs possess generally higher capacitances and E density than EDLCs but often poorer stability and P density (Yu et al., 2015). Diamond-based EDLCs are generally composed of a single diamond material, which has been treated to create a rough or nanoporous surface to increase the electroactive surface area. Diamond-based PCs also benefit from a nanoporous surface structure, but also typically require the fabrication of composite materials with other established PC materials (for example, transition metal oxides or conducting polymers). In addition, for both applications modification of the surface chemistry of diamond plays a significant role in the optimization of the supercapacitor properties.

**FIGURE 1:**
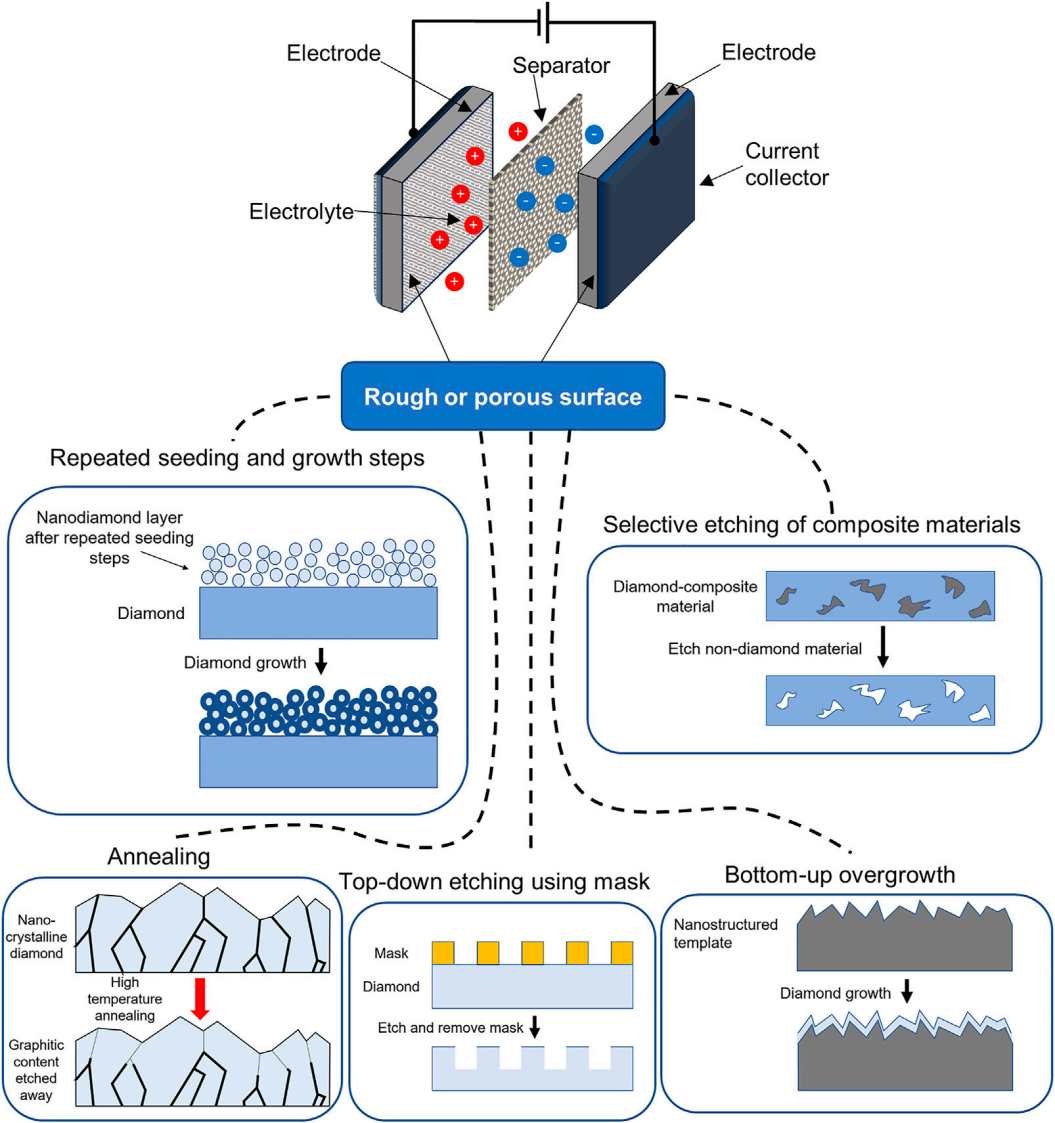
A simplified schematic of the design of a supercapacitor, as well as different methods for creating rough or porous surfaces of diamond for supercapacitor electrodes.

The fabrication methods of diamond-based electrodes with rough or porous surfaces have been comprehensively discussed in previous works (Yu et al., 2021). Briefly, creation of the rough or porous structure has been achieved through either top-down etching, bottom-up overgrowth methods, annealing, selective etching of composite materials, or repeated seeding and growth steps. The top-down etching method utilizes a hard mask such as a metal or metal oxide (Honda et al., 2000, 2001; Yoshimura et al., 2001), and employs an etching technique; for instance plasma etching (Tong et al., 2016), metal particles etching (Ohashi et al., 2009), reactive ion etching (Kondo et al., 2017), or electrochemical etching (Zeng et al., 2019). The bottom-up overgrowth methods make use of a nanostructured template, over which the diamond is grown. These templates include silicon nanowires (Gao et al., 2015; Gund et al., 2015), porous silicon (Ferreira et al., 2005; Silva et al., 2015), ‘black’ silicon (May et al., 2016), silica fibers (Petrák et al., 2017; Vlčková Živcová et al., 2018), silica nanospheres (Gao et al., 2014), anodized aluminum oxide (AAO) (Masuda et al., 2001; Aramesh et al., 2014), and quartz fibers (Kondo et al., 2009). The annealing method applies a high temperature to the diamond in vacuum or an atmosphere of air, pure oxygen, or steam. This has been used to produce onion-like carbon (Portet et al., 2007), or roughened diamond from the etching of graphitic grain boundaries (Ohashi et al., 2011; Kondo et al., 2014; Falahatdoost et al., 2021). The selective etching of diamond composite materials grows diamond embedded in a matrix of sacrificial material such as silicon carbide, which is then removed by a wet chemical etch (Zhuang et al., 2015; Yu et al., 2017). Finally, a porous diamond structure may be created by repeated nanodiamond seeding and chemical vapor deposition growth steps (Wang et al., 2020a). A summary of these fabrication methods is shown in Figure 1, as well as the basic elements of a supercapacitor energy storage device. This includes two electrodes at which charge is stored, the charge-carrying electrolyte, a ion-permeable membrane ‘separator’ to prevent a short circuit, and current collectors connected to an external voltage source.

Diamond has also been combined with conventional PC materials to create diamond-based composite-material PCs. The major advantage of these composite materials is that the PC stability can be improved through using a rigid coating or scaffolding material (Kovalenko et al., 2010; Liu and Li, 2020), while also providing other synergistic effects such as improved P density (Aradilla et al., 2016). The materials used to create diamond-based composites include polymers such as polyaniline (PANI) (Kovalenko et al., 2010; da Silva et al., 2018), polypyrrole (PPy) (Hébert et al., 2015), and poly [3, 4-(ethylenedioxy) thiophene] (PEDOT) (Aradilla et al., 2016); transition metals such as nickel (Shi et al., 2016); and transition metal oxides such as manganese oxide (Yu et al., 2015), nickel hydroxide (Gao and Nebel, 2015), ruthenium oxide (Spătaru et al., 2007), and titanium oxide (Shi et al., 2015). The fabrication methods for these composite materials generally involve either subsequent growth/deposition steps or adding diamond nanoparticles to a polymer synthesis process.

The performance of the diamond-based supercapacitors in past works as measured by gravimetric and areal capacitance are summarized and compared to other conventional EDLC and PC materials in Table 1. Here we have selected works to highlight the performance of diamond-based supercapacitors relative to other state-of-the-art materials. It is clear that currently the gravimetric and areal capacitance values are not as yet competitive with established supercapacitor materials such as activated carbon, graphene, and transition metal oxides. Regarding cycling stability, diamond EDLCs exhibit high performance (similar to other carbon materials) (Yu et al., 2021), while the cycling performance of diamond-composite PCs has been shown to improve that of conventional PC materials (Kovalenko et al., 2010; Gao and Nebel, 2015; Aradilla et al., 2016; da Silva et al., 2018). This suggests that currently diamond-based supercapacitors are best suited for long term and low energy requirement applications, such as micro or planar devices as noted in previous works (Gao and Nebel, 2016).

**TABLE 1:** A summary of the capacitance and lifetime of aqueous-electrolyte-based capacitors using various materials including diamond.

	Material/thickness of porous layer	Gravimetric capacitance (F g^−1^)/Areal capacitance (mF cm^−2^)	Lifetime	References
EDLCs				
Diamond-based	BDD network/selective bulk etching of 3 µm film	13.7/5.19^†^	—	Zhuang et al. (2015)
	N-UNCD/a few nanometers surface etching of 32 µm film	-/28.5	—	Falahatdoost et al. (2022)
Diamond-based (composite)	rGO:ND/drop casting of 300 nm porous film	186/-	∼100% (1,000 cycles, 2 A g^−1^)	Wang et al. (2014)
	CNF:BDD/3.6 µm layer of CNF grown on BDD substrate	-/138	-	Yu et al. (2018b)
Activated carbon	Fully porous, chemically activated corn residue	575/-	65% (20,000 cycles, -)	Gopiraman et al. (2017)
	Activated carbon cloth with approximately 50 nm porous surface	66.9^a^/756	>100% (30,000 cycles, 100 mV s^−1^)	Wang et al. (2015)
Graphene-based	Fully porous laminated graphene theoretical limit	550/-	—	Shao et al. (2015)
	Mesoporous 3D printed graphene oxide with 1.25 mm thickness	20.2/101	—	Yun et al. (2019)
PCs				
Diamond-based (composite)	PAni:BDD:CF composite with nanostructured surface	527/-	—	da Silva et al. (2018)
	CNF:BDD/3.6 µm layer of CNF grown on BDD substrate	-/230	—	Yu et al. (2018b)
RuO_2_	Pressurized RuO_2_:carbon powder	1,390/-	93% (4,000 cycles, 2.5 A g^−1^)	Hong et al. (2014)
	100 µm thick RuO_x_N_y_S_z_ film	-/14,300	80% (5,000 cycles, 10 mA cm^−2^)	Patnaik et al. (2020)
MnO_2_	MnO_2_ powder	1,380/-	—	Toupin et al. (2004)
	α-MnO_2_/γ-MnO_2_ on CNT on yarn	322^a^/3,540	99% (10,000 cycles, 50 mV s^−1^)	Jeong et al. (2019)

As supercapacitor electrode materials are usually designed for either high gravimetric or areal capacitance, examples of both have been provided for each material. BDD, N-UNCD, rGO, ND, CNF, and CF denote boron-doped diamond, nitrogen-doped ultrananocrystalline diamond, reduced graphene oxide, nanodiamond, carbon nanofiber, and carbon fiber respectively.

a The quantities marked with ^a^ were independently calculated using the mass per unit area. All area-dependent quantities refer to the geometric area of the electrodes, not the specific surface area.

While the fabrication of diamond-based EDLCs and PCs have mainly focused on creating a porous surface morphology, we believe that the surface chemistry is another crucial aspect which should be optimized. Diamond is distinct from other carbon-based electrode materials in its high degree of tunability through surface chemistry, affecting the reactivity, surface wettability, and potential window (Siuzdak and Bogdanowicz, 2018). This is largely due to the semiconducting nature of diamond, where the reaction rate depends on the position of conduction and valence band energy levels relative to the electrochemical potential of redox reactions in the electrolyte. For instance, hydrogen terminated diamond causes upward band bending at the surface so that the valence band overlaps with the energy distribution of the oxygen redox couple, increasing the Faradaic reaction rate and producing surface hole conductivity (Chakrapani et al., 2007). On the other hand, oxygen terminated diamond exhibits less band bending and so overlaps less with the energy levels of redox species, leading to lower reactivity and greater capacitive behavior (Chakrapani et al., 2007; Chambers et al., 2020; Falahatdoost et al., 2021). This has important implications for diamond composite pseudocapacitors in particular, where the surface chemistry is thought to play a major role in their operational stability (Kovalenko et al., 2010; Yang et al., 2016). The tunable surface chemistry of diamond is thus a major advantage compared with conventional PC materials; with diamond the surface chemistry offers a method of tailoring the surface properties without affecting the mechanical attributes of the electrode.

The surface chemistry of diamond also affects the electrodes’ potential window in aqueous solution. For instance, oxygen terminated diamond is able to undergo larger operation voltages before electrode-degrading reactions begin to occur compared to hydrogen terminated diamond, while it has further been found that fluorine terminated diamond exhibits a potential window in aqueous solutions as large as 5 V (Ferro and De Battisti, 2003). As discussed previously, a wider potential window leads to greater P and E density for a supercapacitor (Simon and Gogotsi, 2008; Yu et al., 2021). It is also worth noting that diamond with sp^2^ carbon content exhibits a narrower potential window than pure diamond (Martin et al., 1996), so plasma etching of sp^2^ carbon in nanocrystalline diamond is useful for achieving a wider water window. The removal of sp^2^ carbon from the diamond surface has previously been suggested for extending the potential window of diamond for supercapacitor applications (Gao and Nebel, 2016); however, we argue that the chemical surface termination should also be tailored for optimal performance.

In the context of biomedical applications, the surface chemistry of diamond also has a significant impact on its biocompatibility. This property is highly related to the surface wettability, as cells in general are more viable on hydrophilic surfaces (Altankov and Groth, 1994). For instance, the hydrophilic surface of oxygen terminated diamond exhibits greater biocompatibility than the hydrophobic surface of hydrogen terminated diamond (Yang and Narayan, 2019). It is also known that cells prefer rough surfaces, so nanostructured diamond is favorable for cell growth (Yang and Narayan, 2019). Conversely, it has been found that boron-doped diamond cathodically treated in perchloric acid inhibited biofouling (Trouillon and O’Hare, 2010). These are important considerations if diamond-based supercapacitors are to be used in biomedical implants, where cell growth would be encouraged if diamond was used as a device encapsulant, while biofouling resistance would be preferred to allow ions access to the surface of diamond-based electrodes in un-encapsulated devices.

## Assisting Supercapacitor Design Through Computational Modelling

In order to evaluate and optimize the performance of diamond-based supercapacitors, computer simulations are often used to model the electrode-electrolyte interface. In this area, both equivalent electrical circuit models and atomistic models are employed, as well as continuum-level simulations.

Equivalent circuit models involve identifying specific charge transfer processes with electronic circuit elements. The models are fitted to data from electrochemical characterization techniques such as cyclic voltammetry (CV), electrochemical impedance spectroscopy (EIS), and galvanostatic charging/discharging (GCD), from which key parameters can be extracted, such as the capacitance and series resistance (Kondo et al., 2009). Equivalent circuit models are much simpler to implement than atomistic models; however, they provide less explanatory power and are not necessarily applicable to nanoporous materials (Pilon et al., 2015; Zhan et al., 2020a).

Atomistic models account for the nanoscale interactions between the nanoporous electrode and ions in the electrolyte, through molecular dynamics (MD) simulation or Monte Carlo simulation (Simon and Gogotsi, 2013). They assist in elucidating nanoscopic ion transport phenomena within the pores of the electrode, to better understand the processes responsible for high supercapacitor performance. For example, past works have investigated the relationship between pore size and capacitance, exploring the complementary contributions of micro and nano sized pores (Eikerling et al., 2004). Furthermore, other works have analyzed different electrode geometries, such as pores, slits, and fibers, as well as film thickness (Chmiola et al., 2010), and mass loading (Lin et al., 2015).

Regarding the optimization of supercapacitor design, a central challenge is a lack of understanding of the nanoconfined charging dynamics in porous surfaces that give rise to capacitance, something which is not trivial to model (Simon and Gogotsi, 2013; Xiao et al., 2020). To address this issue, previous works have applied MD, continuum-level simulations, and machine learning techniques to investigate nonlinear ion dynamics in porous surfaces (Feng and Cummings, 2011; Jiang et al., 2011; Zhan et al., 2020a). It was found that the capacitance has an oscillatory dependence on the pore size (Feng and Cummings, 2011; Jiang et al., 2011), while nonlinear pore-size-dependent confined ion dynamics can lead to capacitive current peaks which may appear Faradaic (Zhan et al., 2020a). Nevertheless, there is yet to be a detailed explanation and prediction for the power and energy density of supercapacitors whose pore sizes are below the size of the ion solvation shell (Simon and Gogotsi, 2013); a question which is still being investigated by these models (Zhan et al., 2020b).

For diamond-based supercapacitors, these models need to account for the specific properties of diamond electrodes. Here, the effect of surface chemistry is significant and must be considered; for example, chemical surface functionalities significantly change the surface wettability. The redox reactions of diamond-based PCs should also be considered in simulations such as equivalent circuit modelling. Moreover, due to the semiconducting nature of diamond both the space charge region and electrical double layer contribute to the total capacitance of the electrode, and should be incorporated into capacitance calculations, as has previously been done in equivalent circuit models (Falahatdoost et al., 2022). In the context of optimization of the pore geometry in diamond electrodes, it has been previously suggested that anodized alumina oxide (AAO) templates could be used for easily tuning the pore size and shape, film thickness, and mass loading (Gao and Nebel, 2016). This has previously been done to create diamond-like carbon nanotubes (Masuda et al., 2001), and other nanoarchitectures (Aramesh et al., 2014). While current diamond-based supercapacitors have not given particular consideration to the pore geometry, using AAO templates in conjunction with modelling results could provide a tool to enhance the electrode capacitance.

## Discussion

While there has been significant progress in the development of diamond-based supercapacitors, their properties suggest suitability for specific applications. Here it is useful to separately consider diamond-based EDLCs and PCs.

For diamond-based EDLCs, the fundamental disadvantage is that the performance is not currently competitive with state-of-the-art supercapacitor materials in terms of capacitance and energy storage (Yu et al., 2015; Gao and Nebel, 2016). Nevertheless, diamond is attractive for its high durability, biocompatibility, and wide potential window in aqueous media (Gao and Nebel, 2016; Garrett et al., 2016; Wang et al., 2020a). Furthermore, while single crystal diamond involves a high production cost, recent diamond-based supercapacitor electrodes increasingly utilize nanodiamonds or nanocrystalline diamond which can be fabricated at significantly less expense (Williams, 2011; Turcheniuk and Mochalin, 2017). This suggests diamond EDLCs are best suited for small-scale, high-power electronic applications (Gao and Nebel, 2016), such as radiofrequency identification, wireless sensors, or biomedical implants (Gao et al., 2014; Siuzdak and Bogdanowicz, 2018; Wang et al., 2020a). Biomedical applications are also suggested by the diamond electrode’s ability to operate in physiological solution (Garrett et al., 2012; Falahatdoost et al., 2021). This approach has previously been demonstrated with other materials and could be used to power small biomedical devices within the body (Chae et al., 2017; Raravikar et al., 2017; Sim et al., 2018). It has been suggested that supercapacitors can be used in hybrid devices where batteries or energy harvesting devices provide baseload power and supercapacitors can provide peak power (Wang et al., 2020b). For example, a thermoelectric generator in conjunction with a supercapacitor was utilized for powering a pulse oximeter from a person’s body heat (Torfs et al., 2006), and a combination of a kinetic energy harvester and supercapacitor was proposed for wireless sensor applications (Olivo et al., 2010). Supercapacitors have also been shown to be useful as standalone power sources for implantable sensors, for instance in monitoring pH levels (Yamada et al., 2004; Pandey et al., 2011). Other applications could include devices for biosensing such as for EEG, ECG, blood pressure, body temperature, pulse, and glucose levels; or devices for stimulation such as artificial retinae, neurostimulators, and pacemakers (Wang et al., 2020b). Such applications typically require powers of 1–100 µW in standby mode, and up to 30 mW in active mode (Patel and Wang, 2010). This requirement is achievable with the power densities of current diamond-based supercapacitor devices (Yu et al., 2021), which have already demonstrated several working prototypes to operate devices such as LEDs, timers, and fans (Cui et al., 2019; Wang et al., 2020b). While the stiffness of diamond precludes it from situations demanding mechanical flexibility, its high durability and biocompatibility provides an advantage over existing supercapacitor materials for long term applications, such as chronic biosensing.

For diamond-based PCs, the greater E density allows for a greater range of applications. Here, the major advantage is the potential for diamond to be used in composite-material PCs with complementary attributes, to enhance for example the stability by providing a rigid scaffold (Kovalenko et al., 2010; Gao and Nebel, 2015; Aradilla et al., 2016; da Silva et al., 2018). Considering biological applications, the biocompatibility of diamond-composite PCs would partly depend on the properties of the non-diamond material. Of the established PC materials, conducting polymers are generally considered biocompatible, with PAni, PPy, and PEDOT:PSS being employed for supercapacitors in biological applications (Sim et al., 2018; Liu et al., 2021; Ramanavicius and Ramanavicius, 2021). In addition, transition metal oxides such as MnO_2_ and RuO_2_ have exhibited good biocompatibility when the electrode is treated to prevent electrode degradation and ions being eluted into solution (Chae et al., 2017; Wang et al., 2019; He et al., 2020; Shige Wang et al., 2020). Taking this into account, diamond-composite electrodes may assist in the utilization of supercapacitors for long-term biological applications with larger energy requirements. The major drawback of using diamond in these composite electrodes are the added complexity of fabrication (Siuzdak and Bogdanowicz, 2018).

To further improve the performance of diamond-based supercapacitors we believe that additional enhancements can be obtained by fully considering the effect of surface chemistry and utilizing computational simulations to optimize the supercapacitor design for enhanced capacitance and energy storage. In particular, oxygen or fluorine terminated diamond exhibit significantly larger potential windows than as-grown diamond surfaces (Ferro and De Battisti, 2003; Falahatdoost et al., 2021). Furthermore, beyond increasing the surface area of the electrode, computational modelling has found that pore size and shape, film thickness, and mass loading have a large impact on the capacitance and thus E density of the supercapacitor (Simon and Gogotsi, 2013). If the specific surface properties of diamond are taken into account, this modelling can assist in optimizing current diamond electrode fabrication processes and can be tested through adjustable templates such as AAO.

**FIGURE 2:**
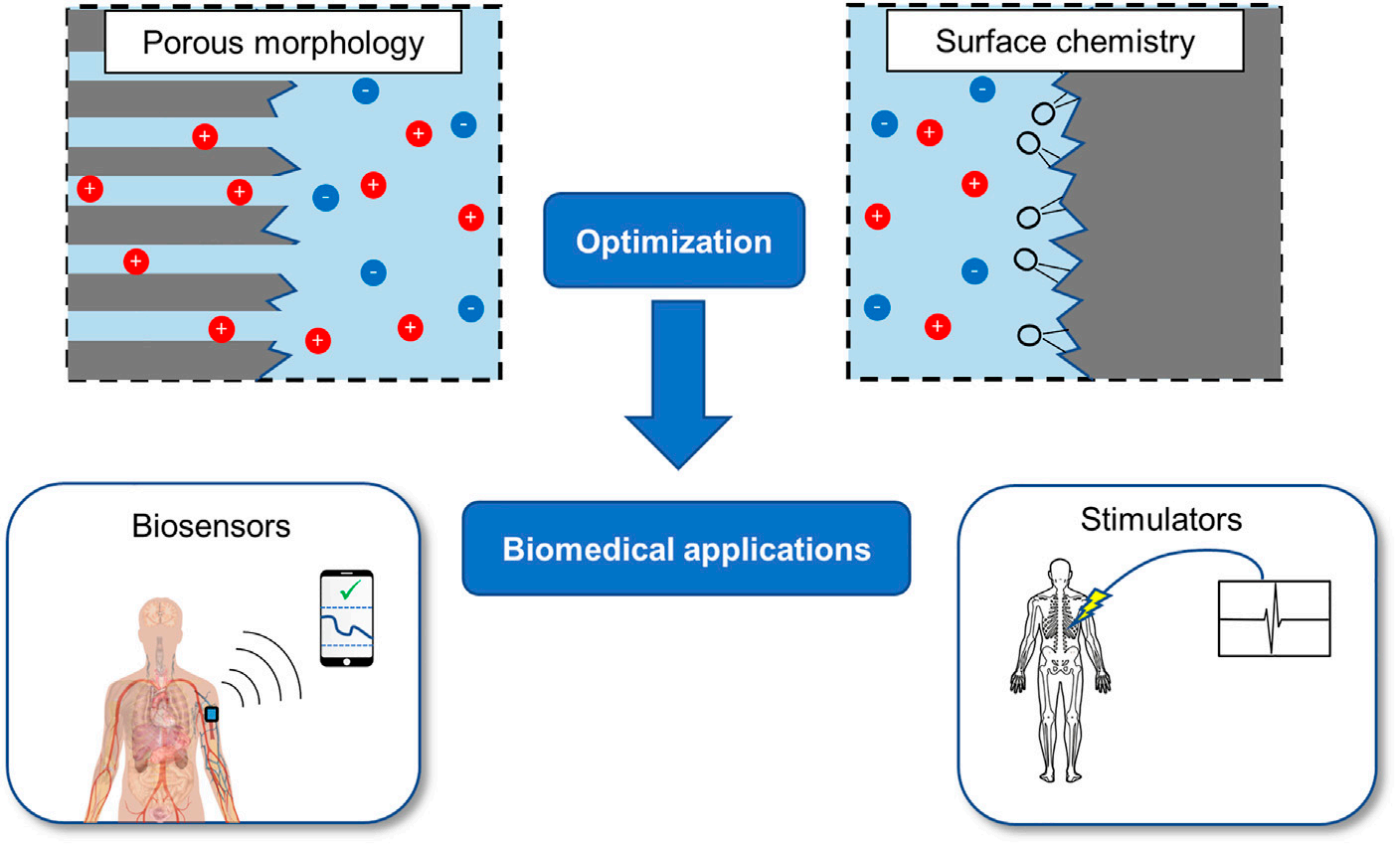
A summary of the areas we have identified for the optimization of diamond-based supercapacitors, as well as the two major categories of implantable biomedical applications that could utilize the advantages of diamond.

To summarize, diamond-based supercapacitors represent a promising avenue for future energy storage solutions. We believe that diamond is well-suited for biomedical applications as outlined in Figure 2; in particular, chronic implantable devices which require intermittent data transfer such as biosensors we believe would benefit from diamond’s durability and biocompatibility. On the other hand, diamond-composite PCs may enhance conventional PCs for cyclic and environmental stability. Both of these types of supercapacitors we argue may benefit from proper consideration of the surface chemistry and design optimization through computational modelling. With these advantages, we believe that diamond-based supercapacitors can make a valuable contribution to energy storage solutions.

## Data Availability

The original contributions presented in the study are included in the article/Supplementary Material, further inquiries can be directed to the corresponding authors.
